# Market-level assessment of the economic benefits of atrazine in the United States

**DOI:** 10.1002/ps.3703

**Published:** 2014-01-21

**Authors:** Paul D Mitchell

**Affiliations:** Department of Agricultural and Applied Economics, University of WisconsinMadison, WI, USA

**Keywords:** AGSIM, conservation tillage, maize, no-till, sorghum, triazine herbicides

## Abstract

**BACKGROUND:**

Atrazine and other triazine herbicides are widely used in US maize and sorghum production, yet the most recent market-level assessment of the economic benefits of atrazine is for market conditions prevalent in the early 1990s, before commercialization of transgenic crops. Grain markets have changed substantially since that time; for example, the size of the US maize market increased by 170% from 1990–1992 to 2007–2009. This paper reports a current assessment of the economic benefits of atrazine.

**RESULTS:**

Yield increases and cost changes implied by triazine herbicides are projected to reduce maize prices by 7–8% and sorghum prices by 19–20%. Projected consumer benefits from lower prices range from $US 3.6 to 4.4 × 10^9^ annually, with the net projected economic benefit for triazine herbicides to the US economy ranging from $US 2.9 to 3.4 × 10^9^ annually because lower prices imply reduced producer income. Productivity gains from triazine herbicides maintain an estimated 270 000–390 000 ha of land in non-crop uses that generate environmental benefits not accounted for in this analysis.

**CONCLUSION:**

Even in the current era, with transgenic varieties dominating crop production, atrazine and the other triazine herbicides continue to be a key part of maize and sorghum production and generate substantial economic benefits. © 2013 The Authors. PestManagement Science published by JohnWiley & Sons Ltd on behalf of Society of Chemical Industry.

## INTRODUCTION

**Figure 1:**
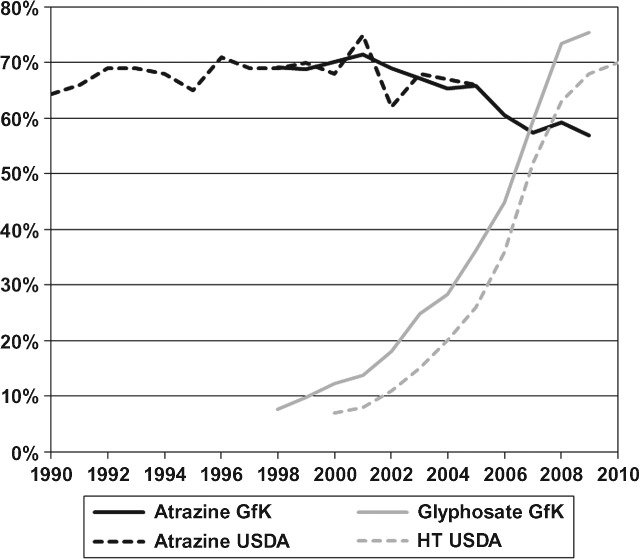
Percentage of US corn acres treated with atrazine, treated with glyphosate and planted with a herbicide-tolerant (HT) variety (Sources: Atrazine GfK and Glyphosate GfK,^52^ Atrazine USDA^74^ and HT USDA^18^).

Triazine herbicides have been used by US crop producers for over 50 years. Atrazine (6-chloro-*N*^2^-ethyl-*N*^4^-isopropyl-1,3,5-triazine-2,4-diamine) was first commercially available for maize (*Zea mays* L.) production in the United States in 1959.^1^ Since then, atrazine has become the most widely used chloro-*s*-triazine herbicide in the United States and the most commonly used maize herbicide for many years, only recently surpassed by glyphosate (Fig. 1). Atrazine is the keystone of herbicide-based weed control in maize and crops such as sorghum (*Sorghum bicolor* L.), sweet maize (*Zea mays* var. *saccharata*) and sugar cane (*Sacharum officinarum* L.).^2^,^3^ In the United States, propazine (6-chloro-*N*^2^,*N*^4^-diisopropyl-1,3,5-triazine-2,4-diamine) is used on sorghum, while simazine (6-chloro-*N*^2^,*N*^4^-diethyl-1,3,5-triazine-2,4-diamine) is used on maize and sweet maize acres and for weed control in many specialty crops such as citrus (genus *Citrus*), grapes (genus *Vitis*) and other fruits and nuts.^2^

Economic assessments of the benefits of atrazine have been part of the research and debate surrounding atrazine. Several studies have examined the farm-level economics of atrazine or have analyzed benefits at larger scales, holding crop prices and planted areas fixed.^4^–^11^ To assess the market-level benefits of atrazine and triazine herbicides, a few studies also included grain supply changes to account for price effects. Carlson^12^ summarized previous comprehensive national or regional economic assessments of the benefits of atrazine and triazine herbicides, but these studies are older, with the most recent published in 1998.^13^

Although more than a decade has passed since these economic assessments were published, they are still in use, in spite of large increases in maize yields, prices and planted area. For example, Ackerman^14^ used the 1994 report of Ribaudo and Bouzaher^15^ as part of an assessment of the economic impacts of a loss of atrazine for growers in the United States. Not only has the underlying demand structure for grain markets changed as a result of population growth and the rapid economic development of nations such as China, but also the overall economic size of the maize market in the United States has increased tremendously since the early 1990s.^16^ The 3 year average planted area of maize in the United States was 31.0 × 10^6^ ha for 1990–1992, and had increased to 35.9 × 10^6^ ha for 2007–2009, a 16% increase.^17^ Yields also increased: the 3 year average maize yield for 1990–1992 was 6.80 Mg ha^−1^ for each ha harvested, and 9.03 Mg ha^−1^ for 2007–2009, or 33% greater.^17^ Average prices received by farmers also increased. The 3 year average maize price was $US 88.20 Mg^−1^ for 1990–1992 and $US 157.11 Mg^−1^ for 2007–2009, a 78% increase.^17^ All combined, the 3 year average total production of maize in the United States increased from 211 × 10^6^ Mg to 325 × 10^6^ Mg over the same period, a 54% increase, and the 3 year average market value of this production increased by 170%, from $US 18.6 × 10^9^ to 50.2 × 10^9^ over the same period. Simply adjusting older estimates of the benefits of triazine herbicides for inflation, in the manner of Ackerman,^14^ ignores this expansion of the US maize market and underestimates the benefits. Rather, previous economic assessments of the benefits of triazine herbicides are outdated because they do not incorporate the large expansion of the maize market.

Other trends also imply that previous benefit assessments of triazine herbicides are no longer accurate for current markets and production conditions. Transgenic crops were first commercialized in 1995 and have become widely adopted since then. For example, 86% of the area planted to maize in the United States in 2010 was some type of transgenic (i.e. insect resistant and/or herbicide tolerant).^18^ Indeed, herbicide-tolerant crops have become so popular among farmers that, in 2007, glyphosate [*N*-(phosphonomethyl)glycine] surpassed atrazine as the most widely used herbicide on maize (Fig. 1). However, glyphosate-resistant weeds now threaten the efficacy of glyphosate, with many farmers aware of the problem and concerned about herbicide-resistant weeds.^19^–^24^ In addition, the biofuel industry has become much larger since these previous studies were completed, causing various adjustments in the agricultural sector as a result of higher demand for maize.^25^–^27^ Given market changes and trends such as these, previous assessments of the economic benefits of atrazine and triazine herbicides are outdated and do not accurately represent the current state.

The AGSIM model^28^ is used to provide an updated economic assessment of the benefits of atrazine to US producers, based on the yield effects reported by Bridges.^29^ Similar to previous studies,^12^ a counterfactual approach is used. Specifically, a non-triazine scenario is developed that describes how yields and costs would change if triazine herbicides were not available, and then the economic benefits of triazine herbicides are estimated as the difference between this non-triazine scenario and a baseline scenario in which triazine herbicides are used.

## DATA AND METHODS

### AGSIM overview

**Figure 2:**
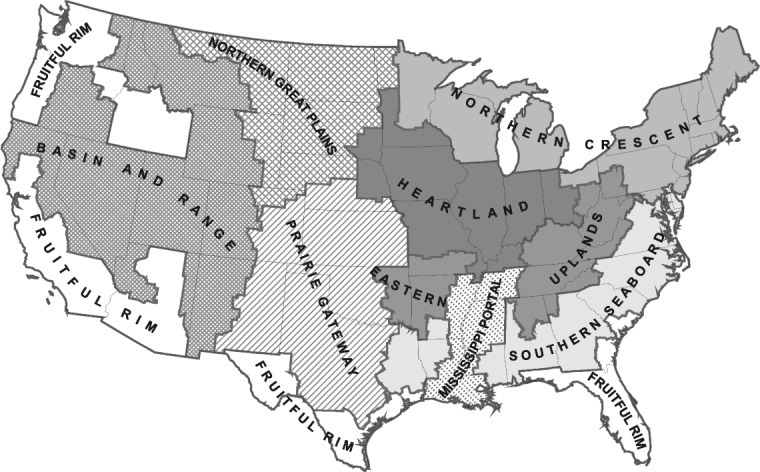
Map illustrating farm resource regions and their agricultural characteristics (Source: USDA-ERS^13^).

AGSIM is an econometric model of supply and demand for US crop production that estimates changes in producer and consumer surplus for different policy scenarios. Taylor^28^ provides a detailed description of AGSIM, and the model is regularly updated to examine new agricultural issues, most recently in 2009 to analyze biofuel policies.^30^ AGSIM models supply and demand, as well as prices and land allocation, for ten major crops in the nine USDA farm resource regions illustrated in Fig. 2.^31^ In addition to maize and sorghum, AGSIM also models barley (*Hordeum vulgare* L.), cotton (*Gossypium hirsutum* L.), oats (*Avena sativa* L.), peanuts (*Arachis hypogaea*), rice (*Oryza sativa* L.), soybean (*Glycine max* L.), all types of wheat (genus *Triticum*) as a single crop, plus dry forage production (‘hay’), which in the United States commonly includes alfalfa (*Medicago sativa* L.), as well as various types of clover (genus *Trifolium*) and/or grasses (family *Poaceae*).

Analyzing policies with AGSIM requires specifying the effect of each policy on average yields and costs for each crop in each farm resource region. Based on these yield and cost effects, AGSIM determines the national market price for each crop, as well as total production and planted area for each crop in each region, after markets and planted areas have moved to a new equilibrium in response to the policy scenario. Differences between scenarios make it possible to estimate how each policy affects equilibrium prices, production and planted area for each crop. Based on these results, AGSIM determines impacts on producer income by crop and region and consumer surplus by crop and by major end use.

AGSIM has a long history and has been used to analyze a variety of agricultural policies, by both academics and government analysts.^28^ TECHSIM, an early predecessor of AGSIM, was used to examine the economics of the loss of pesticides and other pesticide regulatory issues.^32^–^34^ AGSIM has been used to estimate the effects of possible future nitrogen fixation technology for crops and for an *ex ante* assessment of the economic benefits of herbicide-tolerant corn.^35^,^36^ Dinan *et al.*^37^,^38^ used AGSIM to analyze the impact of the Environmental Protection Agency's (EPA) environmental regulations more broadly. AGSIM was used to examine aggregate economic impacts of federal commodity price supports, CRP lands returning to crop production and pesticide use reductions.^39^–^41^ Carlson^12^ and Ribaudo and Hurley^42^ used AGSIM to estimate the economic effects of the loss of atrazine as a weed management tool. AGSIM was also used to analyze the economic benefits of area-wide pest management and to estimate the economic effects of the loss of 2,4-D and phenoxy herbicides.^43^,^44^ The EPA has also used AGSIM to estimate the costs of air pollution regulation and in the most recent atrazine benefit assessment.^45^,^11^ Given its long history of use by academics and by USDA and EPA analysts, AGSIM is well suited for updating estimates of the benefits of triazine herbicides in the United States.

### Scenario description

This analysis uses 2009 as its base year, and the baseline scenario assumes no changes in current yield trends and production costs. Two non-triazine scenarios are defined that project changes in crop yields and costs that would result if triazine herbicides were not available for use in maize and sorghum production in the United States. Differences between these non-triazine scenarios and the baseline scenario indicate how the agricultural economy would change if either of the non-triazine scenarios were realized. Because glyphosate is currently the most widely used herbicide on maize in the United States (Fig. 1), if atrazine and simazine were not available for weed control in maize, an increase in the maize area treated with glyphosate would seem likely, but by how much is unclear. Hence, two non-triazine scenarios were developed to bracket the range of likely farmer responses, following Bridges.^29^

The first non-triazine scenario, ‘increasing glyphosate use for maize’, assumes that, if atrazine and simazine were not available, maize growers could respond by switching to using glyphosate as a substitute herbicide, depending on the weed species in the region and the relative efficacy and cost of alternative herbicides. The projected maize area treated with glyphosate would increase, reaching 100% in all but one region.^29^ The second non-triazine scenario, ‘2009 glyphosate use for maize’, assumes that farmer use of glyphosate on maize will not increase above 2009 levels, even if atrazine and simazine are not available, and instead farmers would switch to non-triazine herbicides other than glyphosate as substitutes. As a result, the percentage of the maize-planted area treated with glyphosate would remain at 2009 levels, but the percentage treated with other non-triazine herbicides would increase. These two scenarios are intended to bracket the likely response of US growers if atrazine and simazine were not available for weed control in maize. Actual farmer responses would probably lie somewhere between the two non-triazine scenarios for maize, so results are reported for both scenarios and interpreted as the range for the expected impact. Note that, as herbicide-tolerant sorghum has not been commercialized, only a single non-triazine scenario is defined for sorghum, a scenario assuming atrazine and propazine are not available for weed control in sorghum.

### Yield and herbicide cost changes

As summarized in Table1, this AGSIM analysis uses the same yield and herbicide cost changes for maize and sorghum as Bridges.^29^ Without triazine herbicides, projected yield losses for maize growers range between 1.4 and 9.6%, depending on the region. Greater yield losses occur for the non-triazine scenario holding the maize area treated with glyphosate at 2009 levels because growers do not increase use of glyphosate, a relatively effective alternative. Sorghum growers experience greater yield losses (more than 20%) because fewer herbicide alternatives exist for atrazine and propazine in sorghum, forcing growers to use less efficacious herbicides. Projected herbicide cost changes for maize and sorghum growers are less than $US 7.50 ha^−1^, with costs actually decreasing in some regions as growers switch to less expensive and/or less effective herbicides. Note that these yield loss and cost changes are averages, and so fail to account for the range of effects that would be experienced by individual growers, and are spread over the entire maize- and sorghum-planted area, not just the area currently treated with triazine herbicides. Furthermore, these cost changes only capture the net change in the cost of herbicide active ingredients; they do not include costs for extra passes over fields to apply herbicides or cost changes resulting from shifts in tillage practices as growers adjust to not using triazine herbicides for weed control.

**Table 1:** Regional yield and herbicide cost changes for maize and sorghum for the two non-triazine scenarios^a^ ^b^

Crop and region	Scenario			
	Increasing glyphosate use on maize		2009 glyphosate use on maize	
	Yield change (%)	Cost change ($US ha^−1^)	Yield change (%)	Cost change ($US ha^−1^)
Maize				
Heartland	−5.26	6.57	−6.04	−0.72
Northern Crescent	−3.45	3.09	−5.23	−6.32
Northern Great Plains	−1.39	2.79	−2.27	1.46
Prairie Gateway	−1.84	0.64	−2.39	−0.57
All other regions	−6.24	4.30	−9.61	0.12
Sorghum	Non-triazine scenario			
All regions^c^	−20.49	−7.39		

a Source: Bridges.^29^

b Yield and cost changes spread over the entire planted area, not just the area treated with a triazine herbicide, and the cost changes do not include additional application costs.

c Yield and cost changes estimated for the Prairie Gateway region and used for all regions.

### Tillage system shifts

Increased adoption of reduced-tillage systems, which reduces soil erosion, is a key benefit of triazine herbicides.^3^,^46^ As a result, the non-triazine scenarios incorporate shifts in aggregate measures of tillage system choices by farmers. Because weeds are a major problem in reduced-tillage systems, herbicides such as atrazine are a key component of weed control in reduced-tillage systems.^46^–^50^ Furthermore, as problems with weeds resistant to glyphosate and other herbicides have developed and spread, academics and extension specialists continue to emphasize the importance of using alternative herbicide modes of action, including triazine herbicide use, to help delay the development of herbicide resistance and to manage weeds resistant to other herbicides.^23^,^51^ As a result, the non-triazine scenarios assume tillage practices will shift towards more intensive tillage, not only for maize and sorghum, but also for soybean and cotton, crops commonly rotated with maize and sorghum in the United States.

Annual tillage system adoption data for maize, soybean and cotton were collected by region from 1998 to 2009 by GfK Kynetec.^52^ Comparable data were not available for sorghum. Tillage system definitions followed the standard classification: conventional tillage is any system with less than 15% of the soil surface covered with crop residue after planting; conservation tillage is any system with 15–30% of the soil surface covered with crop residue after planting; finally, no-till is any tillage system leaving at least 30% of the soil surface covered with residue.^53^

**Figure 3:**
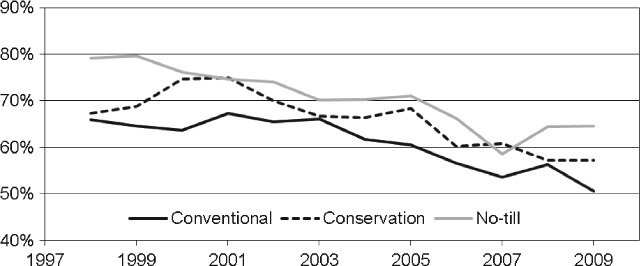
Percentage of conventional tillage, conservation tillage and no-till corn acres treated with atrazine in the United States (Source: GfK Kynetec^52^).

Figure 3 shows the percentage of maize treated with atrazine in conventional tillage, conservation tillage and no-till systems from 1998 to 2009. The declining use of atrazine evident in Fig. 1 is also apparent in Fig. 3. More importantly, Fig. 3 shows the link between reduced tillage and atrazine use: no-till maize had the highest percentage of its planted area treated with atrazine every year, except 2007, and conventional tillage maize had the lowest percentage treated with atrazine. These data show that US maize producers consistently rely on atrazine more in reduced-tillage systems than in conventional tillage systems.

**Figure 4:**
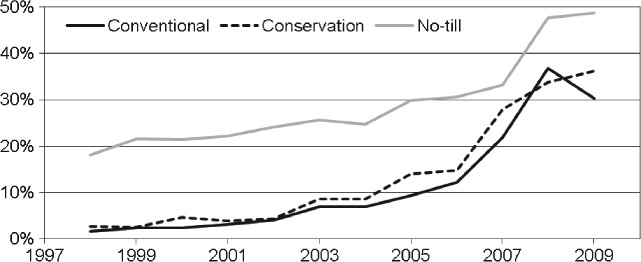
Percentage of conventional tillage, conservation tillage and no-till corn acres treated with both atrazine and glyphosate in the United States (Source: GfK Kynetec^52^).

The link between herbicide-tolerant crops and reduced tillage is well established,^54^–^59^ but the connection between atrazine and no-till maize production remains largely unexplored in the context of widespread adoption of glyphosate-tolerant hybrids. Figure 4 shows the percentage of US maize treated with both atrazine and glyphosate by tillage system from 1998 to 2009. The percentage of maize receiving both atrazine and glyphosate has continuously increased since 1998, following the trends illustrated in Fig. 1. However, a noticeably greater percentage of no-till maize received both atrazine and glyphosate every year, much higher than for conventional and conservation tillage maize. Hence, Fig. 4 demonstrates the continued link between no-till and atrazine use for US maize producers, even in the current era of widespread adoption of glyphosate-tolerant maize.

Table2 reports the 2009 adoption percentages for the three tillage systems used for maize, soybean and cotton in each region. These adoption percentages for the three tillage systems are used as the baseline scenario, with the adoption percentage for maize in the Prairie Gateway region used for sorghum because no data were available. US soybean and maize producers have generally adopted more reduced-tillage systems than cotton growers, and, among the regions, the Prairie Gateway has the highest adoption of reduced tillage for maize and soybean.

**Table 2:** Baseline tillage system adoption percentages by crop and region in 2009

Crop and region	No-till adoption (%)	Conservation tillage adoption (%)	Conventional tillage adoption (%)
Maize			
Heartland	24.3	38.9	36.8
Northern Crescent	25.0	30.9	44.1
Northern Great Plains	41.8	25.9	32.3
Prairie Gateway	46.2	29.5	24.3
Rest of nation	37.2	23.6	39.2
Soybean			
Heartland	46.5	22.8	30.7
Northern Crescent	39.4	27.4	33.3
Northern Great Plains	36.3	22.3	41.4
Prairie Gateway	64.6	14.6	20.8
Rest of nation	48.3	18.1	33.6
Cotton			
Prairie Gateway	10.8	38.7	50.5
Rest of nation	23.4	27.9	48.7
Sorghum			
All regions	46.2	29.5	24.3

For both non-triazine scenarios, the tillage system adoption percentages in Table2 are assumed to shift towards more intensive tillage. Projected adoption percentages for the three tillage systems assume that glyphosate-resistant weeds become an expanding problem and accelerate under the non-triazine scenarios. Without atrazine as a residual herbicide option for maize and sorghum, among the effects would be a shift towards more intensive tillage by farmers to control weeds, especially in maize and sorghum, but also in soybean and cotton, the most common rotational crops following maize and sorghum in the United States. Rather than develop an econometric model to project changes in tillage system choices, a simple approach is used that is based on general trends evident in the data.

A greater shift towards more intensive tillage would occur for maize and sorghum than for soybean and cotton, as chlorotriazine herbicides are not used for weed control in soybean and cotton. Also, as Figs 3 and 4 indicate, no-till maize and sorghum rely more on triazine herbicides (as a preplant and early post-emergence herbicide with residual activity) than conservation and conventional tillage maize and sorghum, and so are more affected under the non-triazine scenarios. This larger effect on use of no-till is assumed to occur for soybean and cotton as well. Finally, farmers will be reluctant to move towards increased tillage owing to cost savings and generally good performance of no-till and conservation tillage systems. As a result, some no-till farmers would move to conservation tillage and some to conventional tillage, and some farmers using conservation tillage would begin to use conventional tillage, but final adoption percentages would probably not differ radically from adoption percentages observed in the last decade. Separate adoption percentages were not used for the two non-triazine scenarios. Also, because the shifts in tillage system adoption percentages are uncertain, three scenarios are developed to capture the range of changes that would probably occur. These scenarios are termed a minor, a moderate and a large shift in tillage system adoption percentages. The percentage point shifts in tillage adoption assumed for these scenarios are based on the subjective assessment of the present author and observed tillage adoption percentages over the last decade.

Table3 reports the projected tillage system shifts assumed to occur under a minor, a moderate and a large shift in tillage practices. For the non-triazine scenarios, these changes are applied to the baseline tillage adoption percentages reported in Table2. For example, the net effect for maize is that planted acres under no-till would decrease by 6.0 percentage points under a moderate shift, but by only 4.5 percentage points under a minor shift and 7.5 percentage points under a large shift. Thus, the adoption percentage for no-till maize in the Heartland will decrease from 24.3% of planted acres under the baseline scenario (Table2) to 18.3% under the non-triazine scenarios for a moderate tillage system shift, a decrease of 6.0 percentage points as reported in Table3. For other regions, initial adoption percentages differ, as reported in Table2, but the shift of 6.0 percentage points as reported in Table3 is applied under a moderate shift.

**Table 3:** Percentage point changes in tillage system adoption percentages for maize, soybean and cotton in the non-triazine scenarios

Crop	Tillage shift	No-till adoption (%)	Conservation tillage adoption (%)	Conventional tillage adoption (%)
Maize	Minor	−4.5	2.0	2.5
	Moderate	−6.0	2.5	3.5
	Large	−7.5	3.0	4.5
Cotton	Minor	−3.5	1.5	2.0
	Moderate	−4.5	2.0	2.5
	Large	−5.5	2.5	3.0
Sorghum	Minor	−4.5	2.0	2.5
	Moderate	−6.0	2.5	3.5
	Large	−7.5	3.0	4.5
Soybean	Minor	−3.5	1.5	2.0
	Moderate	−4.5	2.0	2.5
	Large	−5.5	2.5	3.0

The moderate tillage shift was chosen so that no-till adoption percentages decreased to levels prevalent in 2000–2003. This tillage shift was then allocated so that slightly less than half of it went to a net increase in conservation tillage and slightly more than half went to a net increase in conventional tillage. The tillage system shifts for a minor effect and a substantial effect were chosen so that the no-till decrease was approximately 25% smaller and 25% larger than for the moderate shift, and then the shifts were allocated to increase conservation and conventional tillage as before. These assumptions imply that, under the non-triazine scenarios, tillage system adoption percentages would shift to levels generally similar to those prevailing less than 10 years ago.

### Tillage system cost changes

Tillage systems are defined on the basis of the proportion of the soil surface covered with crop residue after the crop is planted, so that farmers classified within the same tillage system may use different tillage implements and so have different costs. For example, the Conservation Tillage Information Center considers a variety of practices consistent with the term ‘conservation tillage’, including strip-till (both the Midwestern and Southeastern versions), vertical tillage, fluffing harrows, ridge-till and mulch-till.^53^ Because costs for tillage and planting vary across tillage systems, the tillage system shifts for the non-triazine scenarios imply changes in average costs in addition to cost differences due to herbicide changes as reported in Table1.

This analysis estimates the average costs for tillage and planting for each of the three tillage systems by assigning a consistent set of field operations to each system, and then the custom rates or budgeted cost estimates for these sets of field operations are obtained for several states. The cost of planting the crop is also included, because the cost of no-till planting is greater than for conservation and conventional tillage systems. This method captures cost differences between tillage systems (less tillage implies lower cost) while maintaining consistency across states. The supporting information provides a detailed description of the information and process. Using the tillage system adoption percentages in Tables2 and 3 as weights, Table S5 in the supporting information reports the annual average cost ($US ha^−1^) for tillage for the baseline and non-triazine scenarios and the implied cost changes for the non-triazine scenarios relative to the baseline for maize and sorghum, as well as soybeans and cotton. Table4 then combines the cost changes from Table S5 and the herbicide cost changes from Table1 to report the final changes in annual average costs that are due to changes in herbicide use and tillage adoption. Combining two scenarios for glyphosate use on maize with three tillage shift scenarios generates six different non-triazine scenarios, with different cost changes for each. The AGSIM analysis for these six scenarios uses the cost changes in Table4 and the yield changes in Table1. Note that no yield changes are assumed to occur as a result of the shift in tillage practices.

**Table 4:** Change in annual average costs resulting from changes in herbicide use and tillage for the non-triazine scenarios relative to the baseline

	Scenario					
	Increasing glyphosate use on maize ($US ha^−1^)			2009 glyphosate use on maize ($US ha^−1^)		
Crop and region	Minor	Moderate	Large	Minor	Moderate	Large
Maize						
Heartland	8.61	9.36	10.11	1.32	2.07	2.82
Northern Crescent	5.45	6.31	7.17	−3.96	−3.10	−2.24
Northern Great Plains	4.52	5.15	5.78	3.19	3.82	4.45
Prairie Gateway	2.55	3.25	3.94	1.34	2.04	2.73
All other regions	6.81	7.73	8.64	2.63	3.55	4.46
Soybeans						
Heartland	1.94	2.45	2.97	1.94	2.45	2.97
Northern Crescent	2.25	2.85	3.44	2.25	2.85	3.44
Northern Great Plains	1.56	1.98	2.39	1.56	1.98	2.39
Prairie Gateway	1.74	2.20	2.66	1.74	2.20	2.66
All other regions	2.37	3.00	3.63	2.37	3.00	3.63
Cotton						
Prairie Gateway	1.86	2.38	2.90	1.86	2.38	2.90
All other regions	0.80	1.01	1.23	0.80	1.01	1.23
Sorghum						
All regions	−5.48	−4.78	−4.09	−5.48	−4.78	−4.09

## RESULTS AND DISCUSSION

### Crop price and land allocation effects of triazine herbicides

Table5 shows that, relative to the 2009 baseline, the non-triazine scenarios imply noticeably higher prices for maize and sorghum: $US 9.56–11.84 Mg^−1^ for maize and $US 24.53–25.96 Mg^−1^ for sorghum, representing a 7–8% increase for maize and a 19–20% increase for sorghum. All other projected crop price changes for the non-triazine scenarios are typically small, less than 1% relative to the baseline. Across the scenarios, price changes are larger for the non-triazine scenarios that hold the use of glyphosate on maize at 2009 levels because these scenarios impose more restrictions on farmer responses. Price changes vary little across the tillage shift scenarios (minor, moderate, large), implying that the small increases in the cost of production under these scenarios have little effect on market prices. Based on the results in Table5, use of triazine herbicides implies lower crop prices, especially for maize and sorghum, which benefits consumers, but reduces producer income.

**Table 5:** Projected changes in crop price for the non-triazine scenarios

Crop	Baseline ($US Mg^−1^)	Scenario					
		Increasing glyphosate use on maize ($US Mg^−1^)			2009 glyphosate use on maize ($US Mg^−1^)		
		Minor tillage shift	Moderate tillage shift	Large tillage shift	Minor tillage shift	Moderate tillage shift	Large tillage shift
Barley	181.04	0.83	1.00	0.79	1.02	1.00	0.98
Cotton	1397.00	−3.32	−3.25	−3.18	−2.21	−2.14	−2.07
Hay	132.61	−0.14	−0.14	−0.15	−0.14	−0.14	−0.15
Maize	147.32	9.56	9.59	9.62	11.77	11.80	11.84
Oats	161.56	1.55	1.51	1.47	1.76	1.72	1.68
Peanuts	500.94	0.22	0.25	0.28	1.11	1.14	1.17
Rice	259.16	−1.55	−1.55	−1.55	−0.79	−0.79	−0.79
Sorghum	131.61	24.53	24.60	24.67	25.82	25.89	25.96
Soybeans	322.67	−0.21	−0.18	−0.14	−0.33	−0.29	−0.26
Wheat	199.83	−0.55	−0.57	−0.58	−0.45	−0.47	−0.48

Table6 reports the projected planted-area changes by crop for all non-triazine scenarios relative to the 2009 baseline. Given the maize yield decreases and cost changes in Table1 and the price increases in Table5, growers expand the maize-planted area by 359 000 to 444 000 ha, or by 1.0 to 1.2%, depending on the scenario. Even with the much higher sorghum prices implied by the non-triazine scenarios, sorghum acres decrease by 181 000 to 190 000 ha, or by about 6%, because the sorghum yield decrease in Table1 is so large that sorghum becomes less profitable than other crops for many farmers. The other larger projected changes occur for wheat and soybean. The increases in the area planted to wheat range from 64 000 to 73 000 ha across scenarios, while the increases for soybean range from about 17 000 to 45 000 ha. The soybean changes vary more across scenarios owing to the impact of tillage cost changes on relative profitability of soybean. Although sizeable in magnitude, the planted-area changes for wheat, soybean and the other crops are all less than 1%.

**Table 6:** Projected changes in crop-planted areas for the non-triazine scenarios

Crop	Baseline (10^3^ ha)	Scenario					
		Increasing glyphosate use on maize (10^3^ ha)			2009 glyphosate use on maize (10^3^ ha)		
		Minor tillage shift	Moderate tillage shift	Large tillage shift	Minor tillage shift	Moderate tillage shift	Large tillage shift
Barley	1619	−8	−8	−8	−10	−10	−10
Cotton	4170	8	7	7	6	6	5
Hay	24 652	26	26	27	28	28	29
Maize	36 640	369	364	359	444	439	435
Oats	1377	−10	−9	−9	−12	−11	−11
Peanuts	513	0	0	0	−1	−1	−1
Rice	1255	2	2	2	1	1	1
Sorghum	2955	−188	−189	−190	−181	−182	−182
Soybeans	28 745	29	23	17	45	39	33
Wheat	24 089	64	65	66	71	72	73
Total	126 015	292	282	272	392	382	372

Table6 shows that the net effect of these non-triazine scenarios is an increase in the total area planted to crops, ranging from 272 000 to 292 000 ha for the non-triazine scenario allowing glyphosate use to increase on maize, and from about 372 000 to 392 000 ha for the non-triazine scenario holding glyphosate use on maize at 2009 levels. These results imply that current triazine use keeps an estimated 272 000 to 392 000 ha of land in non-crop uses such as pasture or enrolled in the Conservation Reserve Program.^60^,^61^ This economic analysis does not examine the environmental benefits, such as reduced soil erosion and increased wildlife habitat, derived by maintaining this land in non-crop uses. Furthermore, land allocation changes vary more between the two non-triazine scenarios (increasing use of glyphosate on maize and 2009 use of glyphosate on maize) than across the tillage shift scenarios, implying that the yield benefits of triazine herbicides have more impact on land allocation between crops than the small increases in costs under the tillage shift scenarios.

### Producer and consumer effects of triazine herbicides

Table7 reports the projected changes in producer income by crop and by region for the non-triazine scenarios. Projected increases in total farm income range from $US 585 × 10^6^ to 658 × 10^6^ for the non-triazine scenarios with increasing glyphosate use on maize and from $US 1057 × 10^6^ to 1130 × 10^6^ for the non-triazine scenarios holding glyphosate use on maize at 2009 levels. The higher ends of these ranges occur with the minor tillage shift scenario, because the more intensive tillage for the moderate and substantial tillage shifts reduce producer income owing to higher costs. Examining results by crop, the largest positive effect on producer income occurs for maize, ranging from $US 916 × 10^6^ to 1413 × 10^6^, depending on the scenario. These gains are offset by producer income losses from almost all other crops, especially sorghum, soybean, hay and wheat. Although not reported, gains on a per hectare basis for maize range from $US 25 ha^−1^ to $US 38 ha^−1^, while losses for sorghum range from $US 36 ha^−1^ to $US 41 ha^−1^. For all other crops, losses do not exceed $US 10 ha^−1^, except in rice, for which the loss is about $US 12.50 ha^−1^ for the non-triazine scenarios with increasing glyphosate use on maize.

**Table 7:** Projected annual changes in producer income by crop and by region for the non-triazine scenarios

	Scenario					
	Increasing glyphosate use on maize ($US × 10^6^)			2009 glyphosate use on maize ($US × 10^6^)		
	Minor tillage shift	Moderate tillage shift	Substantial tillage shift	Minor tillage shift	Moderate tillage shift	Substantial tillage shift
Crop						
Barley	2.6	2.6	2.5	3.2	3.2	3.1
Cotton	−22.2	−23.2	−24.3	−17.1	−18.1	−19.2
Hay	−45.4	−45.6	−45.9	−54.2	−54.4	−54.7
Maize	952.7	934.5	916.3	1412.6	1394.5	1376.3
Oats	3.0	2.9	2.8	3.1	3.0	2.9
Peanuts	−0.6	−0.6	−0.7	−2.1	−2.1	−2.2
Rice	−15.8	−15.8	−15.8	−9.3	−9.3	−9.2
Sorghum	−111.4	−112.9	−114.4	−99.0	−100.5	−102.0
Soybeans	−62.4	−77.1	−92.0	−66.1	−80.8	−95.7
Wheat	−42.9	−43.2	−43.6	−41.4	−41.7	−42.1
Total	657.8	621.5	585.0	1129.9	1093.6	1057.2
Region						
Heartland	102.5	85.5	68.3	532.8	515.8	498.6
Northern Crescent	183.1	178.5	174.1	193.2	188.7	184.2
Northern Great Plains	168.2	165.4	162.6	194.4	191.6	188.8
Prairie Gateway	336.3	330.7	325.3	443.1	437.6	432.1
All other regions	−132.3	−138.7	−145.2	−233.7	−240.1	−246.5
Total	657.8	621.5	585.0	1129.9	1093.6	1057.2

Examining the results in Table7 by region shows that producer income increases in each of the four specific regions and only decreases in the combined ‘all other regions’. This geographic pattern occurs because the maize income increases in these four main maize production regions offset the income losses for the other crops, except in the combined ‘all other regions’, where producer crop income depends more on crops other than maize. For the non-triazine scenarios with increasing glyphosate use on maize, the greatest gains in producer income occur for the Prairie Gateway region (about $US 330 × 10^6^). However, for the non-triazine scenarios holding increasing glyphosate use on maize at 2009 levels, the greatest gains in producer income occur in the Heartland (around $US 500 × 10^6^), with the Prairie Gateway a close second. This result occurs because projected grower crop allocations show that the change in the area planted to maize in the Heartland is more than twice as large for the non-triazine scenarios holding glyphosate use on maize at 2009 levels than when glyphosate use is allowed to increase. The differences in acreage changes between the two scenarios are not this large for the other crops. Much of this new maize land for the non-triazine scenarios holding glyphosate use on maize at 2009 levels is land reallocated from non-crop uses, so the income increase is larger.

The results in Table7 imply that the yield losses and cost increases that occur without use of triazine herbicides are generally offset by higher crop prices and land reallocations, so that producer income increases for the non-triazine scenarios. This result is not entirely surprising because the non-triazine scenarios are a reverse technological change – farmers revert to inferior weed control technologies. Economic analysis of technological improvements in crop production can find that the value of the yield increases generated by the new technology are eventually more than offset by price decreases resulting from the expanded crop supply as a result of a relatively inelastic consumer demand. Cochrane^62^ was the first formally to conceptualize this process as a ‘technology treadmill’ – in spite of constant adoption of new and more productive technologies, market forces continually erode farm profits.

Recent examples in US agriculture where such results are found include analyses of the market-level benefits of herbicide-tolerant crops and expanded ethanol production. Moschini *et al.*^63^ found that, if yields increase for glyphosate-tolerant soybean, US producers would experience net income losses as a result of lower prices. Similarly, Price *et al.*^64^ found that US producers only capture 4% of the total benefits from herbicide-tolerant cotton, while Fernandez-Cornejo *et al.*^65^ found that herbicide-tolerant soybeans had no significant effect on farm income. Larson *et al.*^26^ analyzed the economic impact of the US ethanol industry on farm income, finding that, if land enrolled in CRP is allowed to convert to crop production, the crop price effects from an expanded grain supply lead to a decrease in aggregate net farm income, even with the increased demand for corn-based ethanol.

Table8 reports changes in consumer surplus for each non-triazine scenario relative to the 2009 baseline, as well as the incidence of these changes – by crop and by end user. Depending on the assumptions regarding the expansion of glyphosate use on maize and the magnitude of the tillage shift, the total loss to consumers under the non-triazine scenarios ranges from about $US 3.6 × 10^9^ to 4.4 × 10^9^ each year. Almost all of these losses fall on maize consumers, a projected $US 3.46–4.27 × 10^9^, which is not surprising, given the large size of the US maize market relative to the other crops and the relatively large price change projected for maize (Table5). Sorghum consumers bear the next largest losses, projected to range from about $US 219 × 10^6^ to 232 × 10^6^ each year, but substantially smaller than for maize consumers because of the much smaller size of the market. The effects are relatively minor for consumers of the other crops.

**Table 8:** Projected annual changes in consumer surplus by crop and end use for the non-triazine scenarios

	Scenario					
	Increasing glyphosate use on maize ($US × 10^6^)			2009 glyphosate use on maize ($US × 10^6^)		
	Minor tillage shift	Moderate tillage shift	Large tillage shift	Minor tillage shift	Moderate tillage shift	Large tillage shift
Crop						
Barley	−4.50	−4.40	−4.29	−5.50	−5.40	−5.29
Cotton	13.2	13.0	12.7	8.82	8.54	8.24
Hay	19.9	21.0	22.0	19.8	20.9	22.0
Maize	−3464	−3475	−3487	−4250	−4262	−4273
Oats	−2.26	−2.21	−2.15	−2.57	−2.51	−2.46
Peanuts	−0.45	−0.52	−0.59	−2.30	−2.36	−2.43
Rice	16.6	16.7	16.7	8.45	8.48	8.51
Sorghum	−219	−219	−220	−231	−231	−232
Soybean	19.0	15.6	12.2	29.5	26.1	22.7
Wheat	34.8	35.6	36.3	28.5	29.3	30.1
Total^a^	−3586	−3600	−3614	−4396	−4410	−4424
End use						
Livestock^b^	−1444	−1448	−1451	−1765	−1768	−1772
Ethanol	−1228	−1232	−1237	−1513	−1517	−1522
Exports	−611	−614	−617	−743	−746	−750
Other^c^	−305	−308	−310	−380	−383	−385
Imports^d^	−2	−2	−2	−5	−5	−5
Total^a^	−3586	−3600	−3614	−4396	−4410	−4424

a Totals may not add owing to rounding and changes in stocks.

b Not including distiller's grain and other ethanol byproducts used as livestock feed.

c Includes food, seed and industrial uses other than ethanol production.

d Consumer surplus loss for importers treated as a gain when summed over all end users.

Examining results by end users, the benefits of triazine herbicides mostly flow to those using large amounts of corn – the livestock and ethanol industries. The livestock industry, largely consisting of beef, hogs, dairy and poultry/eggs, derives the greatest benefit from the triazine herbicides, around $US 1.45–1.77 × 10^9^ annually. These benefit estimates are aggregated across the entire supply chain, summing benefits accruing to livestock farmers, processors/handlers, distributors, retailers and final consumers. Separating this livestock benefit further into the portion accruing to each type of entity along this supply chain requires data and modeling beyond the current capability of AGSIM. Separating this benefit into the portion accruing to each type of livestock is also difficult, as the necessary USDA data are not available and are difficult to develop.^66^

With a consumer surplus benefit of around $US 1.23–1.52 × 10^9^ annually, the ethanol industry is a close second to the livestock industry in terms of the value of the benefits derived from triazine herbicides. Because the livestock industry uses ethanol byproducts as animal feed, and thus is part of the ethanol supply chain, a portion of this estimated benefit also actually accrues to the livestock industry, in addition to the benefits calculated above. Foreign consumers of US grain also derive benefits from triazine herbicides owing to the lower crop prices, especially for maize. In Table8, these consumer benefits are measured by the exports category and range from around $US 610 × 10^6^ to 750 × 10^6^ annually. These benefits are for the entire range of uses for maize and other grains when exported, including livestock feed, as well as food and other industrial uses, and for all consumers along the supply chain, including processors/handlers, distributors, retailers and final consumers. Lastly, US consumers other than the livestock and ethanol industry derive benefits from lower crop prices, ranging in value from about $US 305 × 10^6^ to 385 × 10^6^ annually. These values are for all other uses of maize and the other crops modeled by AGSIM, including food, seed, and any other industrial uses, and again, are for all consumers along the supply chain.

Table9 reports the annual net economic benefit of triazine herbicides by crop. This benefit is calculated as the negative of the annual net change in social surplus (the sum of producer income changes in Table7 and consumer surplus changes in Table8) by crop for each non-triazine scenario. This annual net change in social surplus is the benefit of the non-triazine scenarios relative to the current state, so the negative reverses this change to become the benefit of the current use of triazine herbicides relative to the non-triazine scenarios.

**Table 9:** Projected annual net benefit from use of triazine herbicides by crop

Crop	Scenario					
	Increasing glyphosate use on maize ($US × 10^6^)			2009 glyphosate use on maize ($US × 10^6^)		
	Minor tillage shift	Moderate tillage shift	Large tillage shift	Minor tillage shift	Moderate tillage shift	Large tillage shift
Barley	1.86	1.81	1.76	2.28	2.23	2.18
Cotton	8.92	10.27	11.64	8.26	9.61	10.98
Hay	25.5	24.7	23.8	34.4	33.6	32.7
Maize	2511	2541	2570	2838	2867	2897
Oats	−0.69	−0.66	−0.62	−0.53	−0.50	−0.47
Peanuts	1.02	1.14	1.26	4.38	4.49	4.61
Rice	−0.88	−0.91	−0.95	0.81	0.78	0.74
Sorghum	330	332	334	330	332	334
Soybean	43.4	61.5	79.8	36.6	54.8	73.0
Wheat	8.09	7.67	7.26	12.83	12.41	11.99
Total	2929	2979	3029	3267	3317	3367

The net benefit is positive for almost all crops in all scenarios, meaning the gains in consumer surplus more than offset producer income changes, so that society in net is better off with use of triazine herbicides. Not surprisingly, the largest effects occur for maize, with net gains to society ranging from about $US 2.5 × 10^9^ to almost $US 2.9 × 10^9^ annually across the non-triazine scenarios. Net social gains for sorghum are next largest, at around $US 330 × 10^6^ annually, with gains for soybean the next largest, but still much less than $US 100 × 10^6^ annually over all the non-triazine scenarios. Overall, projected net gains to the US economy from use of triazine herbicides range from $US 2.9 × 10^9^ to almost $US 3.4 × 10^9^ across all the scenarios.

The results in Tables 5 to 8 can also be interpreted as the benefits of triazine herbicides by simply changing the signs of the reported values. Specifically, the results in these tables are the net changes when moving from the 2009 baseline to the specified non-triazine scenario. Thus, by changing the signs of these changes, these results become the effect of shifting from the non-triazine scenario to the 2009 baseline in which US crop producers use triazine herbicides – in other words, the changes when switching from not using triazine herbicides to using them.

Based on Table5, the use of triazine herbicides by US maize and sorghum producers lowers sorghum prices by around $US 24.50 Mg^−1^ to almost $US 26.00 Mg^−1^ and maize prices by around $US 9.50 Mg^−1^ to almost $US 12.00 Mg^−1^, with minimal effects on prices for other crops. Based on Tables7 and 8, these price effects reduce producer income by around $US 0.58–1.13 × 10^9^ annually, but benefit consumers by $US 3.6–4.4 × 10^9^ annually. As reported in Table9, the resulting annual net economic benefit ranges from $US 2.9 × 10^9^ to almost $US 3.4 × 10^9^ for the US economy. Finally, based on Table6, triazine herbicides reduce the total amount of land in the United States that is devoted to crop production by 270 000–390 000 ha, with this land currently in non-crop uses such as planted to grasses or trees as part of enrollment in the Conservation Reserve Program or used for pasture.

## CONCLUSIONS

Triazine herbicides have been used by US crop producers for over 50 years, beginning with simazine for maize in 1958. US producers rely on atrazine, which for many years was the most widely used herbicide for maize production, only recently surpassed by glyphosate in popularity (Fig. 1). Even in the current era of widespread reliance on transgenic crops in the United States, atrazine continues to be popular, used on 57% of US maize acres in 2009 (Fig. 1). Atrazine is also commonly used on sorghum, sweet maize and sugar cane in the United States. In addition, propazine is registered for use on sorghum, and simazine is registered for use on maize, as well as on other fruit and nut crops.

In spite of its continued widespread use, recent economic assessments of the benefits of atrazine and the other triazine herbicides have been lacking, particularly market-level assessments that account for changes in crop prices and planted areas as a result of triazine herbicides. The last market-level assessment was published in 1998.^13^ US markets for maize and other crops have changed much since that time, with large increases in prices, yields and planted areas. This paper presents an updated economic assessment of its benefits for major commodity crops, including maize and sorghum, though not for all crops for which it is registered for use.

The analysis reported here uses AGSIM, an econometric model of supply and demand for ten major US crops that has been widely used to examine a variety of different policies.^28^ AGSIM uses a counterfactual approach that requires specifying how crop yields and costs of production would change for a policy, and then AGSIM projects changes in crop prices and land allocations for each crop, as well as changes in producer income and consumer surplus. The analysis here developed six non-triazine scenarios that projected how US producers would respond to not having triazine herbicides for maize and sorghum production.

Firstly, producers would increase use of non-triazine herbicides as substitutes, with glyphosate probably a popular choice for many growers. However, because glyphosate use has already reached very high levels among US maize producers (Fig. 1), two glyphosate use scenarios are specified to bracket the likely grower response. Following the model of Bridges,^29^ glyphosate use on maize is first allowed to increase as projected, up to 100% adoption, and then glyphosate use is held constant at 2009 levels and growers instead substitute other non-triazine herbicides besides glyphosate, as projected. These shifts in herbicide use imply decreases in maize and sorghum yields and changes in herbicide costs, for which this analysis uses the estimates of Bridges^29^ (Table1).

Secondly, without triazine herbicides, growers would shift towards more intensive tillage, not only because an effective residual herbicide could not be used (atrazine) but also to address growing problems with glyphosate-resistant weeds. Table2 reports baseline tillage system adoption rates by crop and region, and Table3 reports the shifts in these adoption rates under the non-triazine scenarios. Rather than develop a predictive model, the shifts in Table3 used for this analysis assume that tillage adoption would move to levels similar to those common 5–10 years ago in the United States. These tillage shifts would occur not only for maize and sorghum but also to a lesser extent for soybean and cotton, crops commonly rotated with maize and sorghum. Costs for no-till, conservation tillage and conventional tillage systems are estimated for these four crops, using crop budgets from several states (supporting information), and then the net effect of these tillage shifts on the average cost of production is determined for each crop and region (Table S5). AGSIM analysis combines cost changes due to tillage shifts (Table S5) and due to herbicide use changes (Table1) to generate cost changes for six different non-triazine scenarios (Table4).

AGSIM projections for the non-triazine scenarios are reported for crop prices (Table5), crop-planted areas (Table6), producer income changes (Table7), consumer surplus changes (Table8) and the effect on net social surplus by shifting from each non-triazine scenario to the 2009 baseline in which US crop producers use triazine herbicides (Table9), which can be interpreted as the benefit of triazine herbicides.

Based on these results, triazine herbicides lower sorghum prices by about $US 24.50–26.00 Mg^−1^ and maize prices by about $US 9.50 Mg^−1^ to almost $US 12.00 Mg^−1^, with minimal effects on prices for other crops. These price effects reduce producer income by around $US 0.58–1.13 × 10^9^ annually, but benefit consumers by $US 3.6–4.4 × 10^9^ annually, for a net economic benefit ranging between $US 2.9 × 10^9^ and 3.4 × 10^9^ annually. Finally, triazine herbicides reduce the total land area in crop production by 270 000–390 000 ha.

The magnitude of these annual economic benefits from triazine herbicides, $US 2.9–3.4 × 10^9^, are generally greater than for previous studies that also used AGSIM. For example, studies using the CEEPES modeling system relied on AGSIM for their market-level estimate of net benefits from triazine herbicides, with the resulting estimates in the range from $US 0.95 × 10^9^ to almost $US 1.00 × 10^9^.^15^,^42^,^67^–^70^ The last comprehensive study^13^ using AGSIM that is comparable with this assessment found annual benefits ranging from around $US 1.2 × 10^9^ to 1.3 × 10^9^. Differences exist between these studies^12^ and the present study in terms of area treated, yield losses without triazine herbicides and herbicide cost changes, but the most significant change driving these differences is a large increase in the size of the US maize market.

More specifically, the area treated with atrazine has fallen from about 70% of the maize-planted area in the mid-1990s, the base period for the Carlson^13^ study, to 57% in 2009 (Fig. 1). Also, the herbicide cost changes for maize used for the Carlson^13^ study are slightly larger and the maize yield losses slightly smaller than those used in this study.^12^ However, the greatest difference between the studies is the tremendous expansion in the size of the US market for maize during the intervening years. Comparing 3 year averages for 1994–1996 and 2007–2009,^17^ the US area planted to maize increased by 16% and average maize yields increased by 24%, so that total maize production increased by 43%. In spite of this production increase, the average maize price increased by 44%, for an overall increase in average market value of US maize production of 109%, from $US 24.1 × 10^9^ to 50.2 × 10^9^.

The estimated annual net benefit of $US 2.9–3.4 × 10^9^ from use of the triazine herbicides in crop production reported here updates these benefit estimates to reflect the substantial changes in the maize market summarized above. Furthermore, this assessment uses an updated AGSIM model that incorporates shifts in market relationships owing to changes such as the widespread adoption of transgenic crops, the expansion of the US biofuel industry and the increased demand for grain and livestock as a result of the rapid economic growth of nations such as China and India.^16^ Simple adjustments of older estimates of the benefits of triazine herbicides for inflation, as used by Ackerman,^14^ ignore the many changes in markets and policies that have occurred since the mid-1990s.

The version of AGSIM used for this analysis was updated for the 2008 crop year, for which marketing ends in August 2009.^30^ Since that time, commodity prices and planted areas have changed from the 2009 baseline used for this analysis, begging the question as to how these results would change under the market conditions of 2012, with 39.0 × 10^6^ ha of maize planted and substantially higher market prices. In general, AGSIM estimates of price effects and consumer and producer surplus do not change substantially for different price bases, implying that the results reported here still generally apply for current market conditions.^28^ An update would be needed if significant market condition changes occurred, as would be the case for any empirical market model such as AGSIM.

The estimated net benefits reported here do not account for any of the environmental benefits from triazine herbicides that are due to land allocation effects. Specifically, this analysis projects that triazine herbicides reduce the total US land area in crop production by 270 000–390 000 ha. This land is currently in non-crop uses, such as planted to grasses and trees for enrollment in the Conservation Reserve Program or pasture.^60^ Such land probably generates more benefits for wildlife than as crop land, and, as such land is generally more marginal for crop production, is likely to have lower soil erosion rates than if used for crops.^61^,^71^,^72^ Thus, triazine herbicides also generate economic benefits such as wildlife habitat and erosion control. Furthermore, the more intensive tillage projected to occur without triazine herbicides implies additional reductions in soil erosion owing to triazine herbicides, as well as less overall fuel consumption. The benefits of this reduced soil erosion have been estimated to range between $US 210 × 10^6^ and 350 × 10^6^ annually.^73^ As a result, the estimated annual net benefit of $US 2.9–3.4 × 10^9^ for triazine herbicides reported here is in some sense a lower bound on the actual benefits, as it does not include values for these sorts of benefits. Nevertheless, the economic assessment summarized here updates the analysis to a base year of 2009, finding that triazine herbicides generate substantial benefits for the US economy, even without accounting for these environmental benefits.

## Supporting Information

Supporting informationmay be found in the online version of this article.
