# Supplementary material for: Market-level assessment of the economic benefits of atrazine in the United States
Source: Pest Manag Sci. 2014 Jan 21;70(11):1684–96. doi: 10.1002/ps.3703 (PMC4282455; doi:10.1002/ps.3703)
Supplement: Supplementary file 1 — Supplementary [file ps0070-1684-SD1.docx]

Table S1. Cost ($ ha^-1^) for select tillage operations and crop planting in eleven U.S. states

|  |  |  |  |  |  | ------------ Crop Planting ------------ | | | |  |
| --- | --- | --- | --- | --- | --- | --- | --- | --- | --- | --- |
| State | Crop  Year | Chisel  Plow | Tandem  Disk | Field  Cultivate | Strip  Till | Conventional and Conservation | | No-Till | | Source |
|  |  |  |  |  |  | Maize | Soybean | Maize | Soybean |  |
| Illinois | 2010 | $31.62 | $25.44 | $21.74 | $33.59 | $27.42 | $28.16 | $34.09 | $36.06 | Schnitkey *et al*.^S2^ |
| Indiana^*^ | 2008 | $32.81 | $26.17 | $24.91 | --- | $36.48 | $36.76 | $35.73 | $35.49 | Dobbins and Matli^S8^ |
| Iowa | 2010 | $32.85 | $28.65 | $26.80 | --- | $35.07 | $35.20 | $38.78 | $37.91 | Edwards and Johanns^S9^ |
| Kansas^*^ | 2009 | $25.26 | $22.75 | $22.19 | --- | $31.44 | $31.59 | $34.40 | $34.35 | Twete *et al*.^S10^ |
| Kentucky | 2010 | $34.58 | $29.64 | $25.94 | --- | $34.58 | $35.82 | $38.29 | $37.67 | Halich^S11^ |
| Michigan | 2010 | $33.35 | $28.16 | $24.45 | $40.88 | $36.06 | $36.06 | $40.26 | $37.79 | Stein^S12^ |
| Minnesota | 2010 | $22.58 | $21.86 | $13.51 | --- | $26.80 | $26.80 | $31.74 | $31.74 | Lazarus and Smale^S13^ |
| Missouri | 2010 | $33.09 | $28.26 | $29.52 | --- | $31.99 | $32.95 | $35.15 | $35.25 | Plain *et al*.^S14^ |
| Nebraska | 2010 | $27.00 | $24.60 | $24.28 | $38.38 | $38.21 | $32.11 | $36.95 | $33.69 | Jose and Janousek^S15^ |
| Ohio | 2010 | $34.70 | $31.12 | $27.42 | $42.73 | $38.78 | $38.90 | $39.52 | $39.77 | Ward^S16^ |
| Pennsylvania | 2010 | $41.74 | $38.78 | $37.79 | --- | $42.73 | $42.73 | $47.18 | $47.18 | Pike^S17^ |

^*^Costs adjusted to a 2010 base year using the Prices Paid Index for May.^S3,S4^
