# Supplementary material for: Market-level assessment of the economic benefits of atrazine in the United States
Source: Pest Manag Sci. 2014 Jan 21;70(11):1684–96. doi: 10.1002/ps.3703 (PMC4282455; doi:10.1002/ps.3703)
Supplement: Supplementary file 2 — Supplementary [file ps0070-1684-SD2.docx]

Table S2. Estimated cost ($ ha^-1^) for maize and soybean tillage systems, including the cost of planting, by state

|  | ------------------ Maize ------------------ | | | ---------------- Soybean ---------------- | | |
| --- | --- | --- | --- | --- | --- | --- |
| State | No-Till | Conservation | Conventional | No-Till | Conservation | Conventional |
| Illinois | $34.09 | $55.09 | $90.41 | $36.06 | $57.68 | $106.96 |
| Indiana | $35.73 | $61.38 | $103.96 | $35.49 | $61.67 | $120.65 |
| Iowa | $38.78 | $61.87 | $106.95 | $37.91 | $62.00 | $123.50 |
| Kansas | $34.40 | $53.63 | $89.01 | $34.35 | $53.78 | $101.80 |
| Kentucky | $38.29 | $60.52 | $107.45 | $37.67 | $61.76 | $125.98 |
| Michigan | $40.26 | $68.73 | $105.35 | $37.79 | $70.58 | $122.02 |
| Minnesota | $31.74 | $40.31 | $73.46 | $31.74 | $40.31 | $84.75 |
| Missouri | $35.15 | $61.51 | $106.32 | $35.25 | $62.47 | $123.82 |
| Nebraska | $36.95 | $69.54 | $100.59 | $33.69 | $63.60 | $107.99 |
| Ohio | $39.52 | $73.86 | $114.67 | $39.77 | $75.83 | $132.14 |
| Pennsylvania | $47.18 | $80.52 | $140.17 | $47.18 | $80.52 | $161.04 |
