# Supplementary material for: Market-level assessment of the economic benefits of atrazine in the United States
Source: Pest Manag Sci. 2014 Jan 21;70(11):1684–96. doi: 10.1002/ps.3703 (PMC4282455; doi:10.1002/ps.3703)
Supplement: Supplementary file 3 — Supplementary [file ps0070-1684-SD3.docx]

Table S3. Estimated cost ($ ha^-1^) for cotton tillage systems, including the cost of planting, by state

| State | Crop Year | Conventional  Tillage | Budgets | Conservation  Tillage | Budgets | No-Till | Budgets | Source |
| --- | --- | --- | --- | --- | --- | --- | --- | --- |
| Arkansas | 2010 | $57.55 | 1 | --- |  | --- |  | Flanders *et al*.^S6^ |
| Georgia | 2010 | $48.47 | 2 | $17.29 | 1 | --- |  | Smith *et al*.^S18^ |
| Louisiana | 2010 | $73.04 | 1 | --- |  | --- |  | Guidry^S7^ |
| Mississippi | 2010 | --- |  | $60.97 | 3 | $37.54 | 2 | Riley *et al*.^S19^ |
| North Carolina | 2010 | $42.88 | 2 | $26.16 | 1 | --- |  | Bullen *et al*.^S20^ |
| South Carolina | 2010 | $40.31 | 1 | $24.38 | 1 | $11.12 | 1 | Jones^S21^ |
| Tennessee | 2010 | $57.01 | 1 | --- |  | $15.34 | 1 | McKinley *et al*.^S22^ |
| Texas | 2010 | $90.21 | 3 | $67.03 | 11 | $27.02 | 2 | Klose^S5^ |
