# Supplementary material for: Market-level assessment of the economic benefits of atrazine in the United States
Source: Pest Manag Sci. 2014 Jan 21;70(11):1684–96. doi: 10.1002/ps.3703 (PMC4282455; doi:10.1002/ps.3703)
Supplement: Supplementary file 4 — Supplementary [file ps0070-1684-SD4.docx]

Table S4. Estimated cost ($ ha^-1^) for tillage and planting by tillage system, crop and region.

| Crop | USDA Farm  Resource Region | States Included in Average^a^ | ------------- Tillage System ------------- | | |
| --- | --- | --- | --- | --- | --- |
|  |  |  | No-Till | Conservation | Conventional |
| Maize | Heartland | IL, IN, IA, KY, MN, MO, ½NE, ½OH | $36.00 | $58.91 | $99.45 |
|  | Northern Crescent | MI, ½MN, ½OH, PA | $41.02 | $68.78 | $113.19 |
|  | Northern Great Plains | ½MN, ½NE | $34.35 | $54.93 | $87.03 |
|  | Prairie Gateway | KS, ½NE | $35.25 | $58.93 | $92.87 |
|  | All Other Regions | KY, MO, OH, PA | $40.04 | $69.10 | $117.15 |
| Soybean | Heartland | IL, IN, IA, KY, MN, MO, ½NE, ½OH | $35.84 | $59.37 | $115.10 |
|  | Northern Crescent | MI, ½MN, ½OH, PA | $40.24 | $69.72 | $130.50 |
|  | Northern Great Plains | ½MN, ½NE | $32.72 | $51.96 | $96.37 |
|  | Prairie Gateway | KS, ½NE | $34.13 | $57.05 | $103.86 |
|  | All Other Regions | KY, MO, OH, PA | $39.97 | $70.14 | $135.75 |
| Cotton | Prairie Gateway | TX | $27.02 | $67.03 | $90.21 |
|  | All Other Regions | AR, GA, LA, MS, NC, SC, TN | $21.33 | $32.20 | $53.21 |
| Sorghum | All Regions | KS, ½NE | $35.25 | $58.93 | $92.87 |

^a^States with a ½ received half the weight of the other states when calculating the average. See U.S. Postal Service^S23^ for state abbreviations.
