# Supplementary material for: Market-level assessment of the economic benefits of atrazine in the United States
Source: Pest Manag Sci. 2014 Jan 21;70(11):1684–96. doi: 10.1002/ps.3703 (PMC4282455; doi:10.1002/ps.3703)
Supplement: Supplementary file 5 — Supplementary [file ps0070-1684-SD5.docx]

Table S5. Annual average cost ($ ha^-1^) for tillage, weighted by tillage system adoption percentages, for the baseline and non-triazine scenarios and the cost change for the non-triazine scenarios relative to the baseline.

|  |  | ---------- Average Tillage Cost ---------- | | | | ------ Tillage Cost Change ------ | | |
| --- | --- | --- | --- | --- | --- | --- | --- | --- |
|  |  | Baseline Scenario | --- Non-Triazine Scenarios --- | | | --- Non-Triazine Scenarios --- | | |
| Crop | Region |  | Minor | Moderate | Large | Minor | Moderate | Large |
| Corn | Heartland | $68.25 | $70.30 | $71.04 | $71.79 | $2.04 | $2.79 | $3.54 |
|  | Northern Crescent | $81.43 | $83.79 | $84.65 | $85.51 | $2.36 | $3.22 | $4.08 |
|  | Northern Great Plains | $56.71 | $58.44 | $59.07 | $59.70 | $1.73 | $2.36 | $2.99 |
|  | Prairie Gateway | $56.22 | $58.14 | $58.83 | $59.53 | $1.91 | $2.61 | $3.30 |
|  | All Other Regions | $77.13 | $79.64 | $80.56 | $81.47 | $2.51 | $3.43 | $4.34 |
| Soybeans | Heartland | $65.53 | $67.47 | $67.98 | $68.50 | $1.94 | $2.45 | $2.97 |
|  | Northern Crescent | $78.33 | $80.58 | $81.17 | $81.77 | $2.25 | $2.85 | $3.44 |
|  | Northern Great Plains | $63.38 | $64.94 | $65.35 | $65.77 | $1.56 | $1.98 | $2.39 |
|  | Prairie Gateway | $51.96 | $53.69 | $54.16 | $54.62 | $1.74 | $2.20 | $2.66 |
|  | All Other Regions | $77.58 | $79.95 | $80.58 | $81.21 | $2.37 | $3.00 | $3.63 |
| Cotton | Prairie Gateway | $71.47 | $73.33 | $73.85 | $74.36 | $1.86 | $2.38 | $2.90 |
|  | All Other Regions | $34.89 | $35.69 | $35.90 | $36.11 | $0.80 | $1.01 | $1.23 |
| Sorghum | All Regions | $56.22 | $58.14 | $58.83 | $59.53 | $1.91 | $2.61 | $3.30 |
